# Time trends in dyspepsia and association with *H*. *pylori* and work-related stress—An observational study in white collar employees in 1996 and 2015

**DOI:** 10.1371/journal.pone.0199533

**Published:** 2018-06-22

**Authors:** Stefanie Braig, Simon Berger, David Rothenbacher, Stefanie Schmid, Thomas Seufferlein, Hermann Brenner, Dietrich Rothenbacher, Harald Gündel

**Affiliations:** 1 Institute of Epidemiology and Medical Biometry, Ulm University, Ulm, Germany; 2 Department of Psychosomatic Medicine and Psychotherapy, Ulm University, Ulm, Germany; 3 Department of Internal Medicine I, Ulm University, Ulm, Germany; 4 Division of Clinical Epidemiology and Ageing Research, German Cancer Research Center (DKFZ), Heidelberg, Germany; 5 Division of Preventive Oncology, German Cancer Research Center (DKFZ) and National Center for Tumor Diseases (NCT), Heidelberg, Germany; 6 German Cancer Consortium (DKTK), German Cancer Research Center (DKFZ), Heidelberg, Germany; Institute of Social and Preventive Medicine, SWITZERLAND

## Abstract

**Purpose:**

We aimed to describe time trends in functional dyspepsia and the association of dyspepsia-related factors, *Helicobacter pylori (H*. *pylori)* and work-related stress with functional dyspepsia in white collar employees in 1996 and 2015.

**Materials and methods:**

Repeat cross-sectional study conducted in 1996 (n = 190, response rate = 76.1) and 2015 (n = 195, response rate = 40.2) within a health insurance company in South-West Germany. Dyspeptic symptoms measured according to the Rome III criteria, effort-reward imbalance and further work- or dyspepsia-related factors were assessed by self-administered questionnaire. *H*. *pylori* infection as possible factor for dyspeptic symptoms was measured by a ^13^C-urea breath test or an antigen stool test. Kruskal-Wallis tests and multivariable logistic regression models were calculated comparing the upper tertile of dyspeptic symptom scale to the middle and lower tertile.

**Results:**

Mean dyspepsia symptom scores and work-related stress did not differ comparing 1996 and 2015. In bivariate analyses, dyspeptic symptom scores were consistently correlated with sex, age, and using antacids. Further dyspepsia-related factors were smoking and non-leading occupational position in 1996 and non-steroidal anti-inflammatory drugs as well as high effort-reward imbalance in 2015. High intrinsic effort was positively associated with high dyspepsia symptom scores in both studies. Following multivariable adjustment, we observed a consistent association between high intrinsic effort at work and dyspeptic symptoms, although the association was only marginally statistically significant in 1996. Furthermore, a strong association of somatization, only measured in 2015, with dyspeptic symptoms was shown.

**Conclusions:**

Dyspepsia-related factors may have changed throughout the last decades. Nevertheless, although occupational situations might differ, the intrinsic effort is still strongly associated with dyspeptic symptoms.

## Introduction

Dyspepsia is defined as a relapsing and remitting symptom complex originating from the gastrointestinal tract, often without a specific diagnosis [1]. These symptoms are common in the general population with a pooled prevalence of not yet further diagnostically investigated disorders of 20.8% [1]. However, the prevalence varies considerably due to several measurement definitions of dyspepsia [2,3]. The Rome III criteria for functional dyspepsia is one standard for diagnosis and finally 40% of individuals with dyspepsia consult a physician [1]. Although functional dyspepsia is not related to overall survival, it has a negative impact on sick leave and productivity in work, with high absenteeism rate and a considerable reduction of the work productivity [4].

Several factors have previously been shown to be associated with dyspepsia symptoms including sex, age, non-steroidal anti-inflammatory drug use, use of antacids, *H*. *pylori* infection, and smoking [5–7]. In addition to these potentially associated factors, we also described an association between work-related stress and dyspepsia symptoms approximately 20 years ago [8]. This relationship has also been replicated more recently [9,10]. However, relationships between all of these factors and dyspepsia symptoms remain largely unclear.

Furthermore, working conditions and processes have changed considerably over the past decades. This is particularly true on the office environment where workers face an increase in non-routine, heavily knowledge-based working tasks [11]. The intensive use of information technology has resulted in higher information efficiency and intensified communication [12]. Nevertheless, research on the association of work-related psychosocial factors and dyspeptic symptoms is scarce, especially based on time series. Thus, reconsidering the association between work-related stress and dyspepsia symptoms observed in 1996 [8] might provide important new insights. In addition, there is recent convincing evidence that psychiatric comorbidity (i.e., anxiety and depression), and personality (i.e., neuroticism) are associated with dyspeptic symptoms (see review [2] and [13–15]). Studies also investigated the association between stressful life events and the prevalence or the severity of dyspeptic symptoms, e.g., [16]. However, the role of distress, other than anxiety or depression, is rarely analyzed and studies conducted used different outcome definitions or date back to the last century.

In this repeat cross-sectional analysis of data collected at two time points approximately 20 years apart, we aimed to investigate and compare the relationship between potential dyspepsia-related factors, work-related psychological stress and dyspepsia symptoms among white collar workers employed within the same health insurance.

## Materials and methods

### Study population

In 1996, all employees ≥ 18 years of age (n = 276) working at a health insurance company located in Ulm, South-West Germany were invited to participate in a cross-sectional study aiming to assess the factors influencing intestinal health in 1996 (details in [8]). The same protocol was repeated within the same company approximately 20 years later. Due to a merger with a neighboring branch (Biberach, Germany), the company comprised 492 employees at follow-up. Because of pseudonymized data, the number or persons who had already been interviewed in 1996 could not be determined. For reason of comparability, the same questions were used in both surveys including some additional questions in 2015. The comparison of both studies was approved by the Ethics Board of Ulm University (187/14) and written informed consent of each participant was given.

### Questionnaire

#### Dyspeptic symptoms, sociodemographic factors, lifestyle, and medication

At both points in time, participants were asked to fill in questionnaires including questions related to gastrointestinal tract symptoms and workplace-related stress which were distributed throughout the company. Following the Rome III criteria for functional dyspepsia, a symptom score of dyspeptic symptoms (upper abdominal pain, lack of appetite, and early satiety during meals) was calculated summing up the frequency of the aforementioned symptoms that occurred in the last three months (never 0 points, rarely 1, sometimes 2, often 3 points). Scores were recalculated according to the Rome III criteria in the study conducted in 1996. Scores were not calculated for subjects with missing values for any symptom. Subjects were also asked to provide information on sociodemographic characteristics (gender, age coded as 20–29 years, 30–44 years, ≥ 45 years, school education coded as ≤ 9 years, 10–11 years, > 11 years), lifestyle variables (smoking status coded as current, former, never smoker), BMI measured as underweight or normal weight, overweight, obese according to the definition of the World Health Organization, and alcohol consumption (0 g/week, 1–40 g/week, > 40 g/week), intake of antacids and of non-steroidal anti-inflammatory drugs in the past 3 months (each yes, no). In addition, employees were asked whether pharmacological eradication of the *H*. *pylori* infection had taken place (yes, no), which may potentially reduce dyspepsia symptom score.

#### Work-related stress and coping abilities

Work-related stress was assessed by using the effort-reward-imbalance questionnaire [17]. Occupational effort was measured with 6 items that referred to demanding aspects of work; occupational reward with 11 items including financial reward, promotion prospects, job security, and esteem with acceptable Cronbach’s alpha (1996: effort α = 0.68, reward α = 0.71, 2015: effort α = 0.73, reward α = 0.79). The effort-reward imbalance (ERI) score was calculated based on the ratio of the cumulative scale for effort to the cumulative scale for reward, with a correction for the different number of items in the two scales. An effort-reward imbalance was defined as an ERI ratio in the upper tertile of the distribution. The intrinsic component of the model was derived using 29 Likert-scaled items ranging from 1 (low) to 4 (high intrinsic effort) assessing need for approval, competitiveness, and inability to withdraw from work; Cronbach’s α was 0.71 in 1996 and 2015. The overcommitment subscale widely used in research is part of this scale. Missing values were replaced by the mean of the remaining items of the same subscale in case there was one missing value (effort, intrinsic effort), or up to two missing values (reward) in the corresponding scale. Similar to the extrinsic factor, high intrinsic effort was defined as values in the upper tertile of the distribution. This approach was chosen because of our restricted number of subjects indicating ERI > 1. In models comparing different years, the distributions in 2015 were regarded as the reference. Additionally, the employment status (part time, fulltime), and leading occupational position (yes, no) were assessed.

#### Depression, anxiety, and somatization

In the most recently conducted study only, further screening instruments for depression (Patient Health Questionnaire, PHQ-9) [18], anxiety (Generalized Anxiety Disorder, GAD-7) [19], and somatization (Patient Health Questionnaire, somatic symptom scale, PHQ-15) [20] were used. The PHQ-9 includes 9 symptom criteria of depression (e.g., little interest of pleasure in doing things, feeling down, depressed, hopeless), scored as 0 (not at all) to 3 (nearly every day). Internal reliability was acceptable (Cronbach’s α = 0.76). Similarly, GAD-7 is a seven-item questionnaire assessing feelings of nervousness, anxiety, worrying, and troubles to relax, scaled analogous to PHQ-9. Here, Cronbach’s α was 0.79. PHQ-15 is an instrument to identify somatic symptoms, comprising 15 symptoms (e.g., back pain, dizziness, shortness of breath), all of which are cored from 0 (not bothered at all) to 2 (bothered a lot). Internal reliability was Cronbach’s α = 0.73. PHQ-9 and GAD-7 were dichotomized at the cutoff-point ≥ 10 indicating at least mild depression or anxiety, respectively. By excluding the three gastrointestinal symptoms of PHQ, we slightly modified the PHQ-15 score. As there are no established cutoff points for the modified scale, these scores were categorized by tertiles.

#### *H*. *pylori*

Infection status of *H*. *pylori* was determined by ^13^C-urea breath test in 1996 and by stool antigen test in 2015. For details of ^13^C-urea breath test see [8]. In 2015 the employees were asked to collect a stool sample and mail it to our laboratory at the Ulm University as successfully done in other studies [21]. Stool samples were frozen until analysis. The RIDASCREEN femtoLab. *H*. *pylori* enzyme immunoassay (R-Biopharm Darmstadt, Germany) was used to determine presence of *H*. *pylori* antigens in stool. This monoclonal antigen test was used according to the instructions of the manufacturer. It was shown that both the ^13^C-urea breath test and the stool antigen test had sensitivities and specificities close to 100% [22] and results are therefore mostly identical. However, the stool antigen test is not prone to false negative results which may result among patients taking proton pump inhibitors.

### Statistical analysis

Bivariate Kruskal-Wallis tests were used to compare the proportions of employees with dyspeptic symptom scores in the upper tertile according to levels of the covariates. In addition to bivariate analyses, logistic regression models were used to test the independent association between these factors and the dichotomized symptom score (0 = lower or middle tertile, 1 = upper tertile) with the prevalence of a high dyspepsia symptom score. A-prior factors with bivariate p-values < 0.10 were included in multiple logistic regression models. To enhance power, age and BMI were considered continuously in the fully adjusted models. All statistical procedures were carried out with SAS^®^ 9.4 (SAS Institute, Cary, NC, USA).

## Results

In total, about the same number of employees (n = 211 and n = 200) participated in the two studies (overall response was 76.1% in 1996 and 40.2% in 2015). We excluded 14 subjects in 1996 and 4 subjects in 2015, respectively, because they were trainees, supposing different types of work-related stress in employees and trainees. In order to rule out the presence of an organic disease of the gastrointestinal tract further 7 participants in 1996 and 1 participant in 2015 who had a history of peptic ulcer and were not considered for analysis. Mean dyspepsia symptom score was 2.60 ± 2.30, range: 0–8 points in 1996 compared to 2.32 ± 2.00, range: 0–9 points in 2015.

The majority of the respondents interviewed in both surveys were female (74.9% and 85.7%) (Table 1) with a mean age of 33.4 ± 10.9 vs. 42.0 ± 9.2 years. Educational attainment and body weight of the study subjects were higher in the more recently conducted study. Notably, no differences in occupational distress or intrinsic effort in terms of coping ability became evident when comparing both time points (mean effort-reward imbalance (ERI) 0.59; ± 0.25 in 1996 and 0.59 ± 0.26 in 2015, mean intrinsic effort 67.78, ± 8.25 in 1996 compared to 66.63; ± 7.82 in 2015).

**Table 1:** Distribution of dyspepsia symptom score (proportion in upper tertile) according to sociodemographic factors, lifestyle variables, and current *H*. *pylori* infection status in two independent studies conducted 1996 and 2015.

	1996 (n = 190)^a^				2015 (n = 195)^a^			
Characteristics	n	(%)	Dyspepsia symptoms (prop. in upper tertile [%])	p	n	(%)	Dyspepsia symptoms (prop. in upper tertile [%])	p
Sex				**0.037**				**0.027**
Female	140	(74.9)	47.1		162	(85.7)	40.7	
Male	47	(25.1)	29.8		27	(14.3)	18.5	
Age [years]				**0.015**				**0.003**
20–29	95	(50.8)	52.6		21	(11.1)	71.4	
30–44	55	(29.4)	36.4		88	(46.6)	34.1	
≥ 45	37	(19.8)	27.0		80	(42.3)	32.5	
School education [years]				0.57				0.80
≤ 9	40	(21.5)	40.0		13	(6.9)	46.3	
10–11	118	(63.4)	45.8		110	(58.2)	37.3	
> 11	28	(15.1)	35.7		66	(34.9)	36.4	
Smoking status				**0.010**				0.55
Current	39	(20.9)	64.1		28	(14.9)	46.3	
Former	31	(16.6)	35.5		55	(29.3)	38.2	
Never	117	(62.6)	37.6		105	(55.9)	35.2	
BMI				0.30				0.069
Underweight / normal	136	(72.7)	44.1		115	(61.2)	32.2	
Overweight	44	(23.5)	43.1		54	(28.7)	42.6	
Obese	7	(3.7)	14.3		19	(10.1)	57.9	
Alcohol consumption				0.10				**0.031**
0 g/week	73	(39.7)	46.6		65	(35.1)	49.2	
1–40 g/week	48	(26.1)	50.0		54	(29.2)	27.8	
> 40 g/week	63	(34.2)	31.8		66	(35.7)	31.8	
Use of NSAID (past 3 months)				0.57				**<0.001**
No	91	(48.7)	40.7		65	(32.0)	18.3	
Yes	96	(44.8)	44.8		127	(67.9)	45.7	
Antacids use (past 3 months)				**0.015**				**0.015**
No	157	(84.9)	39.5		165	(87.8)	34.5	
Yes	28	(15.1)	64.3		23	(12.2)	60.9	
*H*. *pylori* infection				0.14				0.50
Negative	160	(85.6)	45.0		154	(89.5)	35.7	
Positive	27	(14.4)	29.6		18	(10.5)	27.8	

Bold letters indicate p < 0.05; prop.: proportion; p values of Kruskal-Wallis test for differences between proportions in upper tertile; numbers may not sum up to total n due to missing values on dyspepsia symptoms; NSAID: non-steroidal anti-inflammatory drugs.

^a^Tertiles based on year 2015.

Dyspeptic symptoms were more prevalent in females than males with 47.1% and 40.7% of women scoring in the upper tertile of the dyspeptic symptom score compared to 29.8 and 18.5% in men in 1996 and 2015, respectively. As Table 1 further shows, younger age was consistently linked to higher dyspeptic symptom scores. We also found higher symptom scores in smokers than in never-smokers (64.1% of the smokers in 1996 and 46.3% in 2015 scored in the upper tertile of the dyspeptic symptom score vs. 37.6% and 35.2%). Obesity was linked to a higher symptom score in 2015 only. Furthermore, non-steroidal anti-inflammatory drug use was associated with dyspeptic symptoms (statistically significantly in 2015 only). Conversely, the use of antiacids was clearly associated with higher dyspeptic symptom scores in both surveys. *H*. *pylori* infection was not found to be associated with dyspeptic symptoms at either time point.

Compared to the first study, more participants worked part time in 2015 (14.4% vs. 44.7%, Table 2).

**Table 2:** Distribution of dyspepsia symptom score (proportion in upper tertile) according to work-related factors and psychosocial variables in two independent studies conducted 1996 and 2015.

	1996 (n = 190)^a^				2015 (n = 195)^a^			
Characteristics	n	(%)	Dyspepsia symptoms (prop. in upper tertile [%])	p	n	(%)	Dyspepsia symptoms (prop. in upper tertile [%])	p
Employment status				0.14				0.78
Part time	27	(14.4)	29.6		85	(44.7)	36.5	
Fulltime	160	(85.6)	45.0		105	(55.3)	38.5	
Leading occupational position				**0.022**				0.51
No	149	(79.7)	47.0		158	(83.6)	38.6	
Yes	38	(20.3)	26.3		31	(16.4)	32.3	
Effort-reward imbalance				0.28				**0.004**
1^st^ and 2^nd^ tertile	97	(53.9)	40.2		124	(66.3)	30.7	
Upper tertile	83	(46.1)	48.2		63	(33.7)	50.8	
Intrinsic effort				**0.026**				**0.001**
1^st^ and 2^nd^ tertile	122	(65.3)	36.9		122	(66.0)	29.5	
Upper tertile	65	(34.8)	53.9		63	(34.1)	54.0	
Depression (PHQ-9)		n.a.						**0.006**
< 10					164	(87.2)	33.5	
≥ 10					24	(12.8)	62.5	
Anxiety (GAD-7)		n.a.						**0.016**
< 10					154	(81.9)	33.8	
≥ 10					34	(18.1)	55.9	
Somatization (PHQ-15)		n.a.						**<0.001**
1^st^ and 2^nd^ tertile					161	(86.1)	31.7	
Upper tertile					26	(13.9)	76.9	

N.a.: not available; bold letters indicate p < 0.05; prop.: proportion; p values of Kruskal-Wallis test for differences between proportions in upper tertile; numbers may not sum up to total n due to missing values on dyspepsia symptoms; NSAID: non-steroidal anti-inflammatory drugs.

^a^Tertiles based on year 2015.

Additionally, Table 2 shows the prevalence of high dyspeptic symptom scores according to work-related factors and psychosocial variables. High dyspeptic symptom scores were more prevalent in subjects with no leading occupational position compared to their leading counterparts in 1996 and in subjects with high compared to low ERI in 2015. At both time points, high intrinsic effort was consistently associated with a dyspepsia symptom score in the upper tertile. Notably, the association between symptoms of mental diseases and dyspeptic symptoms, only measured in 2015, was strong with significantly higher symptom scores in those individuals who were depressed (p = 0.006), or suffered from anxiety (p = 0.016), or somatization (p < 0.001).

With regard to *H*. *pylori*, we saw a significant increase of *H*. *pylori* prevalence with age within the study conducted in 1996, albeit the prevalence was quite small (Table 3). Although the respective prevalence was similar in the age categories 18–29 years and 30–44 years in both periods of time, it was much lower in the age category of > 45 years in 2015. Only a low prevalence of pharmacological eradication of *H*. *pylori* was observed in the more recently conducted study.

**Table 3:** *H*. *pylori* infection according to age in two independently conducted studies in the years 1996 and 2015.

	1996				2015				
Age [yr]	n	n _*H*. *pylori*_	(%)	95% CI	n	n _*H*. *pylori*_	(%)	95% CI	Eradication^a^
18–29	95	5	(5.3%)	[0.02; 0.12]	12	1	(8.3%)	[0.00; 0.38]	0
30–44	55	9	(16.4%)	[0.08; 0.29]	81	13	(16.1%)	[0.09; 0.26]	2
> 45	39	13	(33.3%)	[0.19; 0.50]	83	5	(6.0%)	[0.02; 0.14]	4
	p value < 0.001^b^				p value = 0.113^b^				

CI: Confidence intervals; yr: years.

^a^Self-reported history of pharmacological eradication (n = 6).

^b^x^2^ for difference between groups.

Results of the adjusted logistic regression models are displayed in Table 4. According to **Model 1**, high intrinsic effort was positively and independently associated with dyspeptic symptoms (Odds ratio (OR) 1.97, 95% Confidence interval (CI) 0.95–4.09 in 1996 and OR 2.81, 95% CI 1.35–5.84 in 2015). High ERI was associated with higher odds, but the 95% CI included the null value in both time periods. Following inclusion of mood disorders such as depression, anxiety or somatization, which were only available in the study of 2015 (details in Table 4, **Model 2**), the OR for intrinsic effort was attenuated to statistical non-significance (OR 2.17, 95% CI 0.98–4.80). Of the mood disorder variables, somatization showed a strong association but with large confidence interval (OR 6.97, 95% CI 2.09–23.32).

**Table 4:** Adjusted odds ratios (OR) and 95% confidence interval (CI) for having a high dyspepsia symptom score (upper tertile vs 1^st^ and 2^nd^ tertile) according to effort-reward imbalance and intrinsic effort.

	1996		2015	
	OR	(95% CI)	OR	(95% CI)
**Model 1**^a^				
ERI (upper tertile vs. 1^st^ and 2^nd^)	1.47	(0.73–2.95)	1.28	(0.58–2.82)
Intrinsic effort (upper tertile vs. 1^st^ and 2^nd^)	1.97	(0.95–4.09)	**2.81**	**(1.35–5.84)**
**Model 2**^b^				
ERI (upper tertile vs. 1^st^ and 2^nd^)	**-**		0.84	(0.35–2.05)
Intrinsic effort (upper tertile vs. 1^st^ and 2^nd^)	**-**		2.17	(0.98–4.80)
Depression (PHQ-9 ≥ 10 vs. < 10)			1.47	(0.33–6.62)
Anxiety (GAD-7 ≥ 10 vs. < 10)			1.24	(0.37–4.13)
Somatization (upper tertile vs. 1^st^ and 2^nd^)	**-**		**6.97**	**(2.09–23.32)**

CI: Confidence interval, ERI: Effort-reward imbalance; OR: Odds ratio.

^a^Adjusted for sex, age, smoking, BMI, alcohol consumption, antacids, anti-inflammatory drugs, and occupational position.

^b^Adjusted for sex, age, smoking, BMI, alcohol consumption, antacids, anti-inflammatory drugs, occupational position, depression, anxiety, and somatization (depression, anxiety, and somatization were only available in 2015).

## Discussion

We assessed associations of work-related factors, *H*. *pylori* infection, and sociodemographic factors with dyspeptic symptoms measured following the ROME III criteria during 1996 and during 2015. Our results support previously reported associations between several factors including sex, age, and use of antacids. The role of *H*. *pylori* infections was less clear, potentially due to low prevalence of *H*. *pylori*. Interestingly, we observed a consistent association between high intrinsic effort at work (need for approval, competitiveness, and inability to withdraw from work) and dyspeptic symptoms at both time points. This association was attenuated to non-significance after adjustment for psychosocial measures like depression, anxiety, and somatization in 2015.

Notably, despite considerable changes in working environment over the past two decades, no large differences were observed between the two study populations with regard to work-related stress. One reason might be that the subjects included were employees of a health insurance company, potentially working in a relatively secure working environment. Additionally, the recently conducted study covered a high percentage of female part-time workers which may restrict the overall generalizability. However, in our study population, ERI or the intrinsic effort of female part-time workers did not differ from male fulltime workers (data not shown).

A major limitation is the low response rate in the recently conducted study. Subjects with higher work stress might be underrepresented due to low willingness to participate or tight schedules. This assumption is supported by a stress level lying in a lower range [23]. Furthermore, in addition to a general tendency of reduced participation rate in epidemiological studies [24], the collection of a stool sample may have a deterrent effect on the employees’ willingness to participate. However, participation rates have been known to be higher in the exposed than the unexposed and in the affected compared to the healthy population [25]. Therefore, the effect of a potential participation bias is difficult to determine. An analysis stratified by the branch location, with a participation rate of 54.4% in Biberach and 29.3% in Ulm seems to confirm our result. In the Biberach subgroup, as in the overall analysis, we observed higher odds of scoring in the upper tertile of dyspepsia symptom score given high ERI (OR 1.40, 95% CI 0.48–4.05) and intrinsic effort (OR 2.62, 95% CI 0.95–7.20) comparing the upper vs. the middle and lowest tertile following multivariable adjustment for all dyspepsia-related factors (as done in Table 4, Model 1); Ulm: ERI: OR 1.98, 95% CI 0.44–8.98, intrinsic effort: OR 7.06, 95% CI 1.79–27.80.

Obviously, the analyses are based on a non-clinical population not visiting the clinic due to their gastrointestinal symptoms. This will potentially lead to weaker associations between work stress and dyspeptic symptoms than those seen in a clinical population, but otherwise has the advantage of a clear relationship to a working population. Furthermore, due to the restricted number of persons with *H*. *pylori* infections our study might be underpowered to find a relationship between *H*. *pylori* and dyspeptic symptoms. However, we observed a decreasing importance of *H*. *pylori* infection, in line with data showing low prevalence of *H*. *pylori* in young, urban subjects [26]. Furthermore, since only 5% of individuals suffering from dyspepsia symptoms were infected with *H*. *pylori* [27], it may be of lower importance as a risk factor for dyspepsia.

In spite of the limitations, our results underline the existing evidence that anxiety and depression as well as specific personality factors (see review in [2]) are positively associated with dyspeptic symptoms. However, the association of symptoms of depression and anxiety with dyspeptic symptoms was attenuated and lost statistical significance when mutually adjusting for intrinsic effort and somatization in our data. Thus, this points towards a strong impact of work-related coping style on dyspeptic symptoms. Persons with high intrinsic effort are highly ambitious and feel an extensive need of being esteemed by their colleagues or their supervisors. They are known to exaggerate their efforts beyond what is formally expected [23]. The fact that specific personality traits are related to perceived work stress has already been shown e.g., [28], as have relationships between high overcommitment at work and reduced mental health e.g., [29]. These results are in line with studies revealing different non-work-related coping styles in subjects with dyspeptic symptoms compared to their healthy counterparts [30–33], a more action-oriented vs. passive coping style [30] or negative appraisal [31] in persons affected by dyspeptic symptoms. Consequently, these persons with dyspeptic symptoms may profit from interventions on an individual level changing their perception and their management of (work-related) stress. Furthermore, such an intervention may be particularly important as we could show, in line with previous studies e.g., [34], that dyspeptic symptoms are often accompanied by bodily symptoms additionally contributing to reduced work productivity.

Taken together, our study reveals that occupational distress, particularly a critical work-related coping style, and somatization are important determinants of dyspeptic symptoms with implications for treatment strategies. Preventive efforts at the company level as well as psychosocial interventions may be beneficial to reduce sick leave and ensure productivity and competitiveness in the context of the organization.
